# Epicatechin-induced conformational changes in *β*-lactoglobulin B monitored by FT-IR spectroscopy

**DOI:** 10.1186/2193-1801-2-661

**Published:** 2013-12-09

**Authors:** Alessandro Nucara, Paola Maselli, Valeria Giliberti, Marina Carbonaro

**Affiliations:** Dipartimento di Fisica, Università Sapienza di Roma and CNR-SPIN, P.le Aldo Moro 2, 00185 Rome, Italy; CNR-Istituto di Fotonica e Nanotecnologie, Via Cineto Romano 42, 00156 Rome, Italy; Consiglio per la Ricerca e la sperimentazione in Agricoltura - Centro di Ricerca per gli Alimenti e la Nutrizione, Via Ardeatina 546, 00178 Rome, Italy

**Keywords:** *β*-lactoglobulin, Epicatechin, Protein structure, FT-IR spectroscopy

## Abstract

**Abstract:**

The interaction between whey carrier protein *β*-lactoglobulin B and (-)-epicatechin, a major dietary flavonoid with a wide range of health-promoting biological activities, was investigated by Fourier transform infrared spectroscopy in physiological conditions. Amide I spectra of epicatechin - *β*-lactoglobulin complexes, in D_2_O buffer solutions, pD= 6.8, at molar ratios from 0.5:1 to 15:1, were measured by using a cell device specifically created. Changes in secondary structure elements at increasing epicatechin concentrations were quantified. Two different trends were observed for the intensities of *β*-sheet, random coil, and side chain contributions.

At molar ratios ≤2 the *β*-exposed strand contributions (1625 cm^−1^) increased at the expence of the *β*-antiparallel sheet band (1637 cm^−1^). At molar ratios >2 the intensities of both *β* structures slightly decreased. The same behaviour was observed for the side chain contributions (band around 1610 ÷ 1620 cm^−1^). In addition, a conformational transition to a slightly opened structure, followed by aggregate formation at the highest molar ratios, were revealed. The results suggest that binding of epicatechin to *β*-lactoglobulin in physiological conditions occurs at the surface of the protein molecule, resulting in protein dissociation at molar ratios ≤2 with minor changes in secondary structure. This finding provides further evidence for the possibility of successful use of the protein as a carrier of flavonoids, epicatechin included.

## Introduction

Polyphenols are a heterogenous class of secondary metabolites of plant origin active in the defence system, comprising both low molecular weight compounds - flavonoids and phenolic acids - and highly polymerized tannins (M_*r*_ 500÷5000).

Prevention of cardiovascular and neurodegenerative diseases, osteoporosis and, possibly, cancer has been suggested by epidemiological, clinical and animal studies as potential health-promoting effects consequent to regular consumption of polyphenol-rich foods. Reduction of oxidative stress by modulating of cell signaling, gene expression and enzymatic activity are possibly responsible for the biological activity of polyphenols (Sies 2010). However, polyphenol mode of action is only poorly understood so far and novel mechanisms are currently under investigation (Milenkovic et al. 2012).

Bioavailability of polyphenols, as well as of their in vivo metabolites, appears to be low and highly affected by non-covalent interaction with food macronutrients, especially proteins (Carbonaro et al. 2001; D’Archivio et al. 2010). This interaction occurs during technological treatment and gastrointestinal digestion, being responsible for astringency perception (consequent to interaction with salivary proline-rich proteins). Further transformation of polyphenol-protein complexes has been observed to take place in the colon (Selma et al. 2009).

Flavonoids, in particular flavanols (flavan-3-ol) such as catechins, are the most common polyphenols in the human diets. They are characterized by a basic C6-C3-C6 skeleton, with two aromatic rings (A and B) and a heterocycling ring (C) containing one oxygen atom.

Flavonoids have been shown to inhibit allergens, toxins, viruses, bacteria and carcinogens. However, molecular mechanisms for these effects, and the role of flavonoid-protein interaction, remain to be elucidated (Carbonaro and Grant 2005; Cushnie and Lamb 2011). Available data indicated that molecular size, number and disposition of phenolic nuclei and water solubility affect the strenght of flavonoid-protein binding (Jianbo et al. 2011).

Among flavanols, monomeric (-)-epicatechin (EC) is contained in red wine, tea, cocoa products and many fruits (blackberry, cherries, apple, peach, black grapes). This compound has recently reported to prevent cardiovascular disease, diabetes and some cancers (Ellinger et al. 2012; Jimnez et al. 2012). Proteins, notably serum albumin, are possible candidates for its efficient transport in the human body (Pal et al. 2012).

*β*-Lactoglobulin (BLG), the major whey protein from cow milk, is a protein with unknown function and of high interest for the food and pharmaceutical industries, by virtue of its capacity to bind several bioactive compounds: vitamins (retinol, *α*-tocopherol), fatty acids (palmitic acid), polyphenols (Liang and Subirade 2012; Pervaiz and Brew 1985). Binding of ligands to BLG may occur either in the internal cavity, primary site for hydrophobic molecules, with a high affinity (binding constant *K*_*a*_ ≈5×10^7^ M^−1^ for retinol), or in external sites of the protein, with a lower affinity (Liang and Subirade 2012). Secondary binding sites include a pocket in the groove between the *α*-helix and the *β*-barrel, a site near to the aperture of the *β*-barrel, the outer surface near Trp19-Arg124 and the monomer-monomer interface (Sawyer et al. 1998). *K*_*a*_ values in the range 10^3^−10^4^ M^−1^ have been found for flavonoids, whereas binding number is about 1 mol per mole of BLG for most ligands (Kanakis et al. 2011; Zorilla et al. 2011). Contrasting results are present in the literature regarding ligand binding to BLG in low affinity sites. In particular, under physiological conditions, EC has been reported to bind to BLG with a binding constant *K*_*a*_=3.2×10^3^ M^−1^, and a stoichiometry of 0.9 mol per mole of BLG (Kanakis et al. 2011). However, no binding of EC to BLG have been observed by other authors in physiological conditions (Riihimäki et al. 2008).

Few studies have been performed on structural modifications induced by flavonoid binding to food proteins (Bi et al. 2004; Kanakis et al. 2011). This information may be useful for clarifying flavonoid mechanism of action and for improving their bioavailability and bioefficacy as nutraceutical compounds through efficient delivery by food proteins.

Analysis of amide I contributions in the infrared spectra has been demonstrated to provide very useful information on secondary structure of proteins of food relevance, as well as about their modifications induced by ligand binding or technological treatments, especially when proteins with a high *β*-sheet content are analyzed (Carbonaro et al. 2012). Interaction between catechins and BLG at low molar ratios has been reported to increase *β*-sheet and *α*-helix content and, therefore, to influence the structural stability of the protein (Kanakis et al. 2011). On the other hand, no changes in the secondary structure consequent to catechin binding to BLG at molar ratio ≤ 2.0 have been detected by other investigations. A concentration of catechin ten times higher than that of BLG was necessary to point out significant changes in structural elements induced by catechin binding (Zorilla et al. 2011).

In the present study, interaction of EC to BLG was analyzed at a molecular level by Fourier Transform Infrared spectroscopy (FT-IR) in physiological conditions. The aim of this work was to detect subtle changes of BLG secondary structure as a function of the EC concentration in the range from 0.5:1 to 15:1 EC-BLG molar ratios.

Spectra of BLG and EC-BLG solutions were measured by using a suitable cell device, specifically created by photolithographic technique. Possible nutritional consequences of the binding and potential of BLG as carrier of nutraceutical compounds were discussed.

## Materials and methods

### Materials

EC (E4018), BLG from bovine milk (L8005, B variant, purity ≥ 90%), and deuterium oxide (D_2_O, 99.9% deuterium) were provided by Sigma-Aldrich Chemical Co. (St. Louis, MO, USA) and used without further purification. All other reagents were of analytical grade.

### Sample preparation

BLG was freshly prepared by dissolving powder in 10 mM potassium phosphate/D_2_O buffer, pD = 6.8, at a concentration of 2 mM (36.8 mg/ml). EC was prepared in D_2_O buffer at 1 mM, 2 mM, 4 mM, 10 mM, 15 mM, 20 mM, 25 mM and 30 mM. Protein and flavonoid concentrations were checked spectrophotometrically (DU 640 UV/VIS spectrophotometer, Beckman Coulter Inc. CA, USA) using an extinction coefficient of 17600 M^−1^ cm^−1^ for BLG (M_*r*_=18400) at 280 nm (Liang and Subirade 2010), and 4290 M^−1^ cm^−1^ at 270 nm for EC (Pelillo et al. 2004).

Each EC concentration was added dropwise to the BLG solution to have a polyphenol: protein molar ratio (*mr*) of 0.5:1, 1:1, 2:1, 5:1, 7.5:1, 10:1,12.5:1 and 15:1 with respect to the 1 mM protein. These mixtures were used as such for FT-IR analysis, after a time of 2 hours necessary for the formation of the EC-BLG complexes. All procedures were performed under a nitrogen atmosphere.

### Infrared measurements

**Figure 1:**
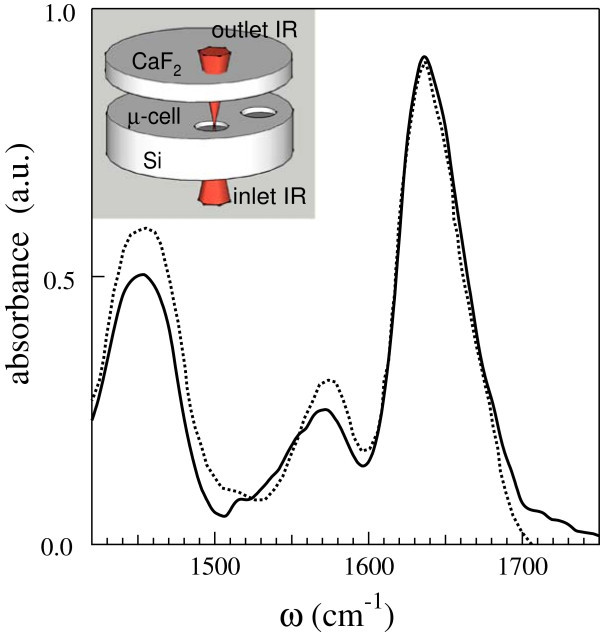
**The amide I and II bands of 1 mM BLG in D**_**2**_ **O buffer solution as obtained by the 9** ***μ*** **m thick** ***μ*** **-cell.** The data are reported as a continuous line. The absorption spectrum of 1mM BLG from Ref. 10 is also reported as dashed line. In the inset a schematic drawing of the cell device is shown.

Absorption spectra in the region of the amide I were acquired using a cell expressly created by a photolithographic technique. The cell consists on a 1 cm thick silicon window with cilindrical wells carved on its top surface (well’s depth from 1 to 9 *μ*m). Photolithography has been used to transfer a geometric pattern from a photomask to a 1.7 *μ*m layer of photoresist (1811) on the window surface, and subsequently the silicon has been removed through dry etching with Solfur Exafluoride (SF_6_). Once filled with the solutions, the wells, hereafter *μ*-cells, were sealed with a CaF_2_ window (see inset of Figure 1). Spectra were collected with resolution of 2 cm^−1^ at 24°C by means of an infrared microscope (Bruker IRscope) coupled with a FT-IR interferometer (Bruker IFS 66V).

Since a thin film of solution wet the silicon surface outside of the wells thus altering the effective thickness of the solution within the *μ*-cell, we retained only spectra with identical integral value of the transmitted intensities for the succeeding analysis.

The *μ*-cells prevent the use of polimeric spacers for samples thickness, yielding to a higher spectra reproducibility and to a significant saving of protein (the typical amount of solution is 1 *μ*l). Moreover, samples of different thickness can be exploited in a unique experiment without removing the cell from the microscopy stage. Interference fringes at the silicon-solution and solution-CaF_2_ interfaces affect the spectra from the *μ*-cells: these artifacts can be subtracted by a fitting procedure.

## Results and discussion

In Figure 1 are shown the amide I and II absorption bands obtained with the 9 *μ*m depht *μ*-cell. The spectrum, corrected for the interference fringes, is compared with that available from the literature for 1 mM BLG in D_2_O buffer solution (Dong et al. 1996), in order to estabilish the reliability of our procedure. The band centered around 1460 cm^−1^ includes both the amide II and the DHO bending absorptions. Residual intensity of the amide II modes is observed around 1560 cm^−1^, where side-chain contributions are also expected. The most intense amide I absorption is observed around 1640 cm^−1^: its lineshape is in good agreement with that reported in the literature, when scaled by an appropriate factor. It is worth noticing that the relative intensities of the absoption peaks are almost identical for the two spectra in Figure 1.

**Figure 2:**
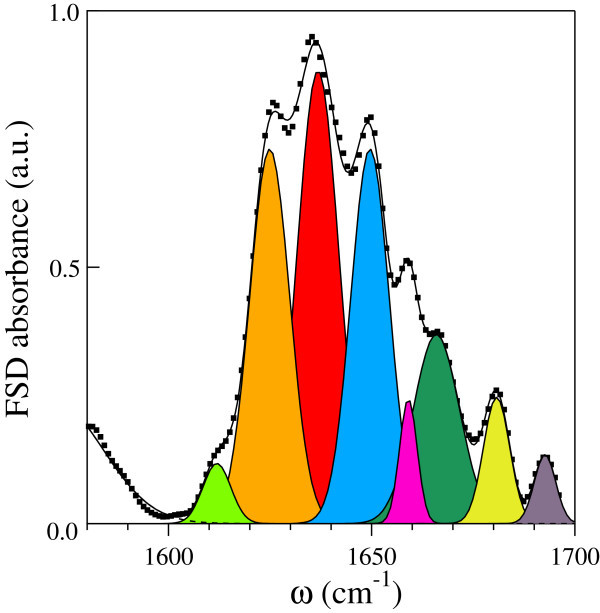
**The Fourier-Self- Deconvolved (FSD) spectrum of 1mM BLG in D**_**2**_ **O buffer solution.** The FSD parameters used in the procedure are Bandwidht = 5 cm^−1^ and Resolution Enhancement Factor = 2. The experimental FSD data are reported as filled squares. The best fit of the FSD spectrum is also reported as a continuous line together with the single Gaussian contributions.

The amide I spectrum of 1mM BLG has been deconvolved into single components by using Fourier-Self-Deconvolution (FSD) methods and then fitted with Gaussian lineshapes. The FSD spectrum and the best-fit contributions are reported in Figure 2. By adopting a conventional procedure, we assigned the relative area W_*i*_ of the *i-th* Gaussian peak to each secondary structure percentage, assuming identical extinction coefficients for the secondary structure components. In Table 1 the values of W_*i*_ are reported together with the central frequencies of the Gaussian fit.

**Table 1:** **Peak frequencies (first column) and corresponding spectral weights (second column) of the amide I components of 1 mM BLG in D**_**2**_ **O buffer solution**

***ν***_***i***_cm^***−1***^	W_***i***_	Assignment
1612	0.03	side chains/intermolecular aggregates
1624	0.19	exposed *β*-strands
1636	0.35	antiparallel *β*- sheet
1650	0.19	unordered
1659	0.04	*α*-helix
1666	0.13	turn
1681	0.05	*β*- sheet/turn
1693	0.02	*β* sheet/intramolecular aggregates

The error on the peak frequency is ± 2 cm^–1^, while that on the spectral weight is ± 0.01.

The two Gaussians centered at 1624 and 1636 cm^−1^ account for most of the amide I intensity (54%) and they are both assigned to *β*-sheet secondary structure contributions. Actually, the central frequency of the antiparallel *β*-sheet occurs between 1632 ÷ 1640 cm^−1^ (Barth 2007), depending on the H-D exchange rate between protein and solvent. The contribution centered at 1624 cm^−1^ has been attributed to “exposed” *β*-strands, i.e. to aminoacid residues hydrogen-bonded with the solvent. The relative intensities of these two *β* contributions depend on the monomer-dimer equilibrium of the protein: it has been observed that pH induced monomer-to-dimer formation results in the increase of the antiparallel contribution at 1636 cm^−1^ at the expense of the exposed *β*-strands (Casal et al. 1988).

The small peak (4%) at 1659 cm^−1^ is centered at the vibrational energy of the *α*-helix complexes (Prestrelski et al. 1991).

An intense (19%) absorption due to unordered structures is observed at 1650 cm^−1^. The peaks around 1666 cm^−1^ is usually assigned to the turn structures bridging the antiparallel *β*-sheets, while the band centered at 1681 cm^−1^ may be attributed to the high-frequency antiparallel *β*-sheet resonance as well as to turn structures (Dong et al. 1996). The Gaussian peak centered at 1693 cm^−1^ is ascribed to absorption from *β*-sheet structures involved in intramolecular aggregates. Some authors pointed out that this band originates from aminoacid groups hidden to the H-D exchange (Gomaa et al. 2013), an argument not in contrast with the presence of internal *β* aggregates.

The weak contribution at 1612 cm^−1^ may be due to side-chain absorption as well as to intermolecular *β*-sheet aggregates. A similar contributions has been observed in the amide I absorption band for a number of food proteins (Carbonaro et al. 2008).

Several Gaussian peaks display wide broadenings, suggesting the presence of unresolved structures. However, attempts to further deconvolve the amide I band yield to noisy FSD spectra, unsuitable for data analysis. It must be noted that the W_*i*_ values reported in Table 1 are in excellent agreement with the secondary structure percentages avalaible from the literature.

**Figure 3:**
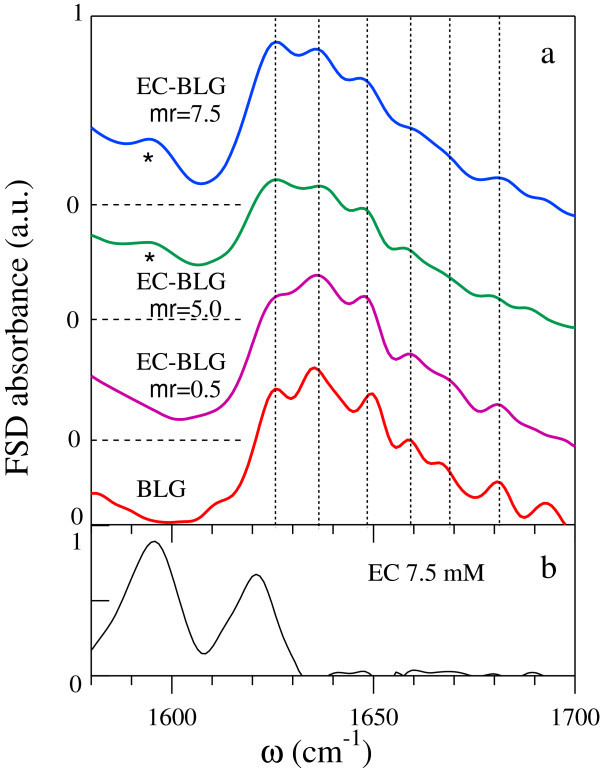
**FSD spectra of the EC-BLG complexes at selected** ***mr*** **and of epicatechin in the Amide I region.** In the panel **(a)** the vertical lines correspond to the central frequencies of the most intense contributions reported in Table 1. The star marks the presence of the EC absorption band at 1595 cm^−1^. In the panel **(b)** the FSD absorption spectrum of 7.5 mM EC in D_2_O buffer solution is shown.

Selected representative spectra of the EC-BLG complexes at different values of *mr* are reported in panel (a) of Figure 3. As *mr* increases, the overall shape of the amide I absorption significantly changes, making the BLG secondary structure assessment more difficult. Indeed, the fine structure of the amide I band becomes less resolved at *mr*>2, so that the weakest bands previously assigned to *α*-helix, turns and to the high frequency *β*-sheet modes are scarcely distinguishable. In particular, the *β* contribution around 1693 cm^−1^ is not observed at any *mr*. Moreover, a remarkable increase of intensity below and around 1600 cm^−1^ is observed in the spectra of the complexes at *mr*>2.

In order to obtain reliable outcomes on the secondary structure of BLG in the complexes, we measured the absorption spectrum of bare EC in D_2_O buffer (panel b of Figure 3). The EC absorption spectrum displays two main peaks at 1595 and 1620 cm^−1^, corresponding to the vibrational modes of C=C aromatic ring, which overlap with the amide I BLG absorption. Other EC bands, detected around 1520 and 1460 cm^−1^ and assigned to C-OH stretching modes, do not affect the intensity of the amide I bands but contribute to the observed increase of intensity at low frequency.

**Figure 4:**
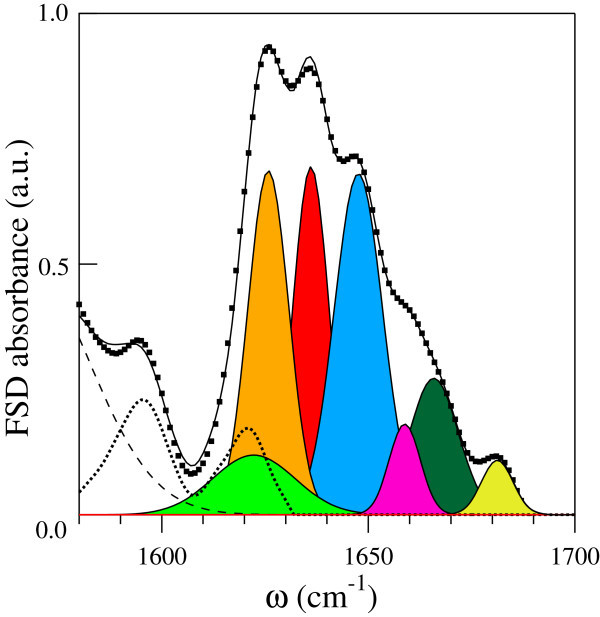
**FSD amide I spectrum of the EC-BLG complex at** ***mr*** **= 7.5.** The experimental FSD data are reported as filled squares and the continuous line is the best fit to data with Gaussian lineshapes. The single secondary structure contributions are reported as filled curves, together with the EC bare spectrum (dotted line). The dashed line at low frequency is the tail of the amide II absorption band.

The EC absorption spectrum, properly scaled for the *mr* values, has been subtracted from those of the EC-BLG complexes during the Gaussian fit analysis, assuming that the BLG and the EC absorptions inchoerently add. We remark that the EC bands affect the BLG spectrum only for *mr*>2. In Figure 4 the Gaussian fit for the complex with *mr*=7.5 is reported. Besides to an overall broadening of the secondary structure contributions, we observe a marked increase of the low-frequency side chains/intermolecular aggregate band and almost identical peak intensities for the *β*-sheet contributions at 1624 and 1636 cm^−1^.

In Table 2 are reported the spectral weights W_*i*_ of the Gaussian contributions for all values of *mr*, according to the bands assignment proposed for BLG.

**Table 2:** **Spectral weights of the secondary structure components of the BLG protein in the EC-BLG complexes as obtained from the spectral deconvolution of the amide I band**

***mr***	***W***_***side−chains/aggregates***_	***W***_***β−exposed***_	***W***_***β−antiparallel***_	***W***_***unordered***_	***W***_***α***_	***W***_***turn***_	***W***_***β−sheet/turn***_
	1607***÷*** 1619	1624***÷***1626	1636***÷*** 1638	1647***÷*** 1649	1658***÷*** 1660	1665***÷*** 1668	1679***÷*** 1681
0.5:1	0.05	0.24	0.30	0.20	0.05	0.14	0.02
1:1	0.06	0.26	0.26	0.23	0.04	0.12	0.03
2:1	0.07	0.28	0.24	0.21	0.03	0.11	0.06
5:1	0.09	0.29	0.23	0.20	0.05	0.11	0.03
7.5:1	0.07	0.25	0.21	0.28	0.05	0.11	0.03
10:1	0.07	0.25	0.20	0.25	0.05	0.11	0.07
12.5:1	0.12	0.23	0.20	0.26	0.04	0.11	0.04
15:1	0.11	0.22	0.19	0.27	0.04	0.13	0.04

The error on the spectral weight is ± 0.01. The wavenumber intervals reported for each component are expressed in cm^–1^.

**Figure 5:**
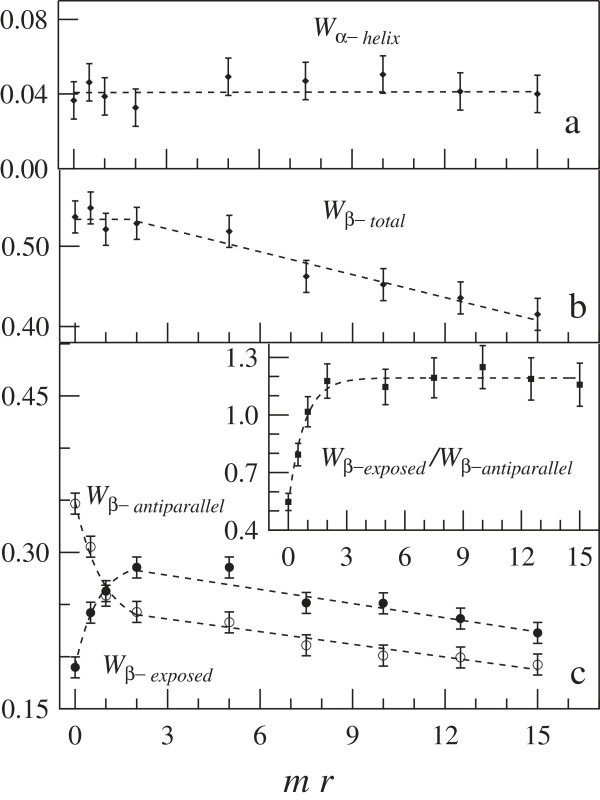
**Spectral weights of BLG secondary structures as a function of the molar ratio.** The ***α*** and ***β*** structures are reported in **(a)** and **(b)**, respectively. In **(c)** the spectral weights of *β*-exposed and *β*-antiparallel structures are shown in the inset of **(c)** the ratio between intensities of the exposed and antiparallel *β* structures is reported.

We first discuss the changes of *β* and *α*-helix structures of BLG as a function of the *mr* value. The spectral weights of the *α*-helices structures are reported in panel a of Figure 5: no significant dependence on the EC content is observed within the accuracy of the data for this parameter. Conversely, *W*_*β*−*total*_=*W*_*β*−*antiparallel*_+*W*_*β*−*exposed*_ (shown in panel b of Figure 5) is constant only for *mr*≤2, but linearly decrease with a slope of -0.009 *mr*^−1^ at the highest molar ratios. These outcomes are not in accordance with previous results on hydrated films (Kanakis et al. 2011), where an increase of both *β*-sheet and *α*-helix secondary structures has been quoted at *mr* = 2. This discrepancy may be due to differences between the structure of BLG in D_2_O buffer solution at pD = 6.8 and that assumed by the protein in hydrated films.

In the panel c of Figure 5 are reported the spectral weights *W*_*β*−*exposed*_ and *W*_*β*−*antiparallel*_ as a function of *mr*. Two different behaviours are detected for these intensities: up to *mr*≤2, the intensity of the exposed *β*-strands increases at the expense of the antiparallel *β* structures, while at the highest *mr* values both intensities linearly decrease with identical slopes. At the lowest *mr* values, these spectral weights can be described by exponential laws, *W*≈*exp*(−*mr*∗*c*), being 1/*c* the rate of increase (decrease) of the *β*-exposed (*β*-antiparallel) structure. A fitting to data provides *c*≈1 for both spectral weights, and their asymptotic values hold 0.24 and 0.29 for *β*-antiparallel and *β*-exposed structures, respectively.

The present findings indicate that significant changes of the BLG *β*-structures occur at concentration of epicatechin close to 1:1 molar ratio and they saturate as *mr* approches the value of 2: within this range, the intensity of the *β*-structures shows the converse behaviour respect to that observed in the pH induced monomer-to-dimer formation ((Casal et al. 1988; Qin et al. 1998) and references therein). Therefore the increse of the intensity of the exposed *β*-strands at 1625 cm^−1^ at the expense of the *β*-antiparallel band provides evidence for BLG dimer dissociation induced by the EC interaction.

At the highest *mr* values both *W*_*β*−*antiparallel*_ and *W*_*β*−*exposed*_ linearly decrease, departing from their respective asympotic values. This behaviour is ascribed to protein denaturation induced by polyphenol excess, finally resulting in aggregation.

In the inset of panel c of Figure 5 is shown the *β*-exposed to *β*-antiparallel ratio at the different *mr* values. A threefold increment is measured up to *mr*=2, whereas at the highest values of *mr* the ratio remains constant within errors. These findings confirm that the crossover between *β* structures occurs at low molar ratios: further addition of EC in the solution only affects the total amount of *β* structure.

**Figure 6:**
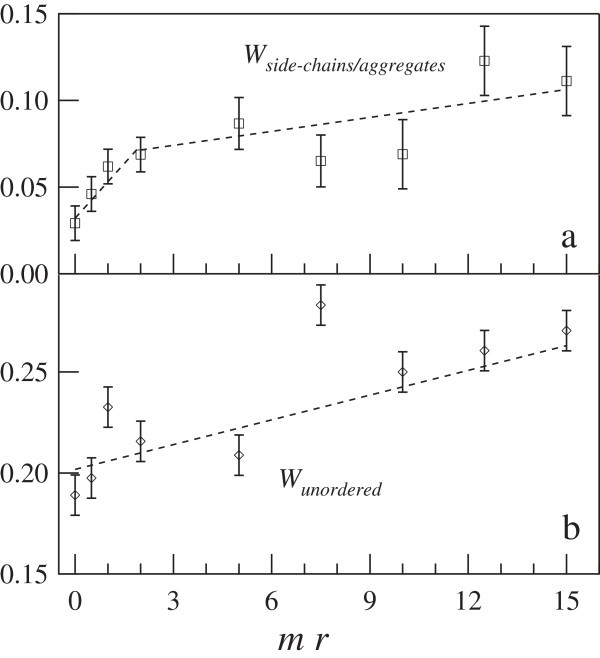
**Spectral weights of BLG secondary structures as a function of the molar ratio.** **(a)**: side-chain/aggregates secondary structures as a function of the *mr*. The errors on the data at *m* *r*>2 have been overestimated in order to account for the overlapping with the epicatechin spectrum. **(b)**: unordered structures reported as a function of the *mr*.

Evidences for the two-regime response to epicatechin addition are observed also in the spectral weight of the side-chains/aggregates contribution, reported in the panel a of Figure 6. Similarly to *W*_*β*−*exposed*_, *W*_*side*−*chain*/*aggregates*_ increases with *mr* changing its slope around *mr*=2. Therefore, we assume that epicatechin-induced dimer dissociation enhances the C=C streching contributions of the aromatic side chains. The smooth linear trend observed for *W*_*side*−*chain*/*aggregates*_ at the highest *mr* values is likely due to formation of molecular aggregates, which are known to contribute in the range 1610 ÷ 1620 cm^−1^ in most proteins. In the panel b of Figure 6 the spectral weight of the unordered secondary structures is reported: here, in spite of a more pronounced data scattering, an enhancement of the random coil moieties is clearly observed.

As a member of the lipocalin family, BLG can bind a variety of ligands, not only retinol but also other hydrophobic or amphiphilic small molecules such as polyphenols. Besides to the large central cavity, at least three different external binding sites have been suggested to be available on the protein surface, showing lower binding affinities. Binding of resveratrol at the protein surface, near Trp19-Arg124, has been suggested (Liang and Subirade 2012). Molecular docking and dynamics simulation studies have indicated that while quercetin and quercitrin were bound to the central cavity, rutin was bound to the entrance of the calyx (Sahihi et al. 2012).

As far as catechins is concerned, although they are suggested to bind in the internal site of BLG on the basis of docking studies, binding constants in the range of 2.2×10^3^ ÷ 1.3 ×10^4^ M^−1^ indicated a low affinity interaction, similar to that measured for binding of ligands to external sites (Kanakis et al. 2011). Other studies suggest that binding of several phenolic compounds is more likely to occur to the surface of the protein than in the internal site (Riihimäki et al. 2008).

The results of the present study show that EC induces concentration-dependent changes in BLG secondary structure at pD = 6.8, mainly consisting in alterations of *β*-sheet structure and in an increase in side-chain spectral contributions and in random coil conformation. Such changes demonstrate that the interaction of EC with bovine BLG B results in protein dissociation at *mr*≥2 and in destabilization at higher *mr* values, finally responsible for protein aggregation. Binding of EC to BLG is suggested to occur at the protein external surface, possibly at a site in the proximity of the dimer interface (residues from 145 to 153, comprising I-strands), thus causing its dissociation into monomers, as monitored by the increase in the percentage of *β*-exposed at *mr*≤2. Minor changes in secondary structural elements are detected at this stage. Notwithstanding, an intermolecular event induced by EC binding, involving small conformational changes, was detected by FT-IR analysis. This is in agreement with the involvement of one exposed *β*-strand in the BLG dimer formation, proposed on the basis of X-ray crystallographic results (Papiz et al. 1986). Moreover, breaking of the intermolecular *β*-sheet hydrogen bonds at I-strands has been found to markedly destabilize the BLG dimer, due to the small molecular interface which involves only a few number of residues (about 6% of the total surface area) (Konuma et al. 2013; Sakurai and Goto 2002). Dimer to monomer dissociation of BLG has also been reported upon interaction with epigallocatechin-3-gallate at neutral pH (Zorilla et al. 2011). It is worth noting that other hydrophobic molecules operate in a similar way at the BLG dimer interface (Konuma et al. 2013).

Further binding of EC to BLG induces changes in secondary structure towards unfolding and aggregation (see panel b of Figure 6). However, the protein maintains a high degree of structural integrity, in agreement with the reported stability of the globular structure of BLG under several conditions.

BLG is known to maintain its conformation during the gastrointestinal digestion. Further protection towards proteolysis by gastric and pancreatic enzymes upon polyphenol binding has been observed (Stojadinovic et al. 2013). Therefore, stoichiometric interaction between EC and folded BLG monomers in physiological conditions may be of biological relevance and worthy of exploitation for nutraceutical applications.

The present information may add insights in planning strategies for successful employ of *β*-lactoglobulin in nutraceutical delivery systems, as well as for increasing beneficial vs adverse properties of this protein.
